# Isotope-Based Analysis of Modified tRNA Nucleosides Correlates Modification Density with Translational Efficiency**

**DOI:** 10.1002/anie.201203769

**Published:** 2012-10-04

**Authors:** Caterina Brandmayr, Mirko Wagner, Tobias Brückl, Daniel Globisch, David Pearson, Andrea Christa Kneuttinger, Veronika Reiter, Antje Hienzsch, Susanne Koch, Ines Thoma, Peter Thumbs, Stylianos Michalakis, Markus Müller, Martin Biel, Thomas Carell

**Affiliations:** Center for Integrated Protein Science at the Department of Chemistry, Ludwig-Maximilians-Universität MünchenButenandtstrasse 5–13, 81377 Munich (Germany) E-mail: thomas.carell@cup.lmu.de Homepage: http://www.carellgroup.de; Center for Integrated Protein Science at the Department of Pharmacy, Ludwig-Maximilians-Universität MünchenButenandtstrasse 5–13, 81377 Munich (Germany)

**Keywords:** isotopic labeling, mass spectrometry, RNA modification, translation, tRNA

Transfer RNAs (tRNAs) are adapter molecules needed to translate genetic information into a peptide sequence.[1] At the ribosome, the anticodon of each tRNA reads the corresponding codon of the messenger RNA. This anticodon–codon interaction allows the ribosome’s large subunit to catalyze amide-bond formation between the cognate amino acids present at the 3′ terminus of aminoacyl-tRNAs and the growing peptide chain.[2] The tRNA adapters required for this process display a surprisingly large chemical diversity.[3] Aside from the four canonical nucleosides A, C, G, and U, more than 100 modified nucleosides are key constituents (Figure 1).[4] The most diverse and complex chemical structures are found in the anticodon stem-loop either in the anticodon at the wobble position or directly adjacent to the 3′ position of the anticodon,[5] suggesting that here the chemical complexity is necessary to establish translational fidelity.[6] The ribosome seems to need the modified anticodon region to better distinguish correctly base-paired tRNA from mispaired interactions in order to prohibit, for example, codon-slippage processes that would lead to frameshifts.[7]

**Figure 1:**
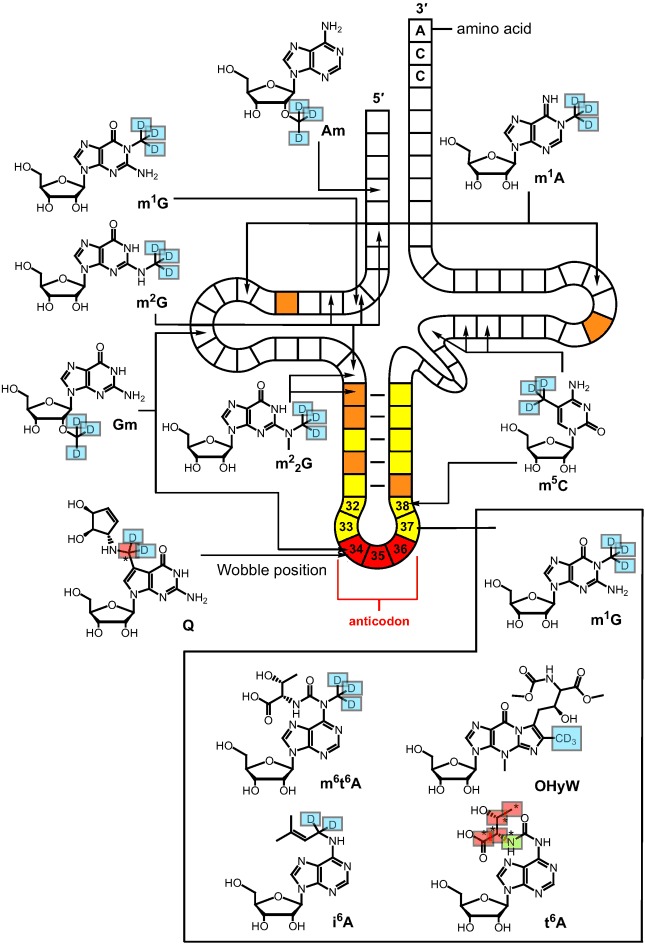
Isotope-labeled tRNA nucleosides present in eukaryotic tRNA and positions where these modifications are typically found. The introduced isotope labels are marked in color: D in a blue box: deuterium; * in a red box: ^13^C; * in a green box: ^15^N. The anticodon is highlighted in red, the remainder of the anticodon stem-loop is in yellow, and positions of Ψ are marked in orange. Abbreviations are explained in Table S1 in the Supporting Information.

In order to investigate how the set of nucleoside modifications influences the translational efficiency we quantified the tRNA modifications individually in various tissues by an isotope-dilution-based LC–MS method. (Details on the materials and methods are given in the Supporting Information, Table S1, and Figure S1). The quantified modification levels were correlated with the translational efficiency by means of an in vitro translation system. For the experiments, 11 representative tRNA modifications (Figure 1) were chemically synthesized as isotope-labeled derivatives.[8] A majority of the investigated nucleosides are located inside the extended anticodon,[9] the other synthesized modified nucleosides are found at various other positions.[10]

As biological material for the analysis we chose a range of different organ tissues from mouse and pig. Porcine tissues were used because they are available in large amounts, while murine tissues were analyzed at a later stage to confirm the results in a genetically more defined organism. For pig, 5–10 g of tissue from two animals was sampled from each organ, while murine samples were obtained from two sets of five animals of which whole organs were analyzed. After total tRNA extraction and complete enzymatic hydrolysis to nucleosides, a mixture of the isotope-labeled tRNA modifications was added and the solution was subjected to LC–MS analysis. The ratios of the mass peak integrals from natural to isotope-labeled nucleosides were determined and calibration curves, which were previously measured for each investigated modified nucleoside, then allowed exact parallel quantification of the respective modifications (see Figure S2 in the Supporting Information).[8a] LC–MS quantification was performed at least in triplicate and results were averaged for each tissue. The error margin of the experiment was in this way limited to around 5 %.

The obtained quantitative values for mouse and pig samples are shown color coded in Figure 2 A and B, respectively, together with the approximate positions of the measured modifications in the tRNA sequence. The values represent the measured number of each modification per 1000 tRNA molecules (‰) (exact values are listed in Tables S2–7 in the Supporting Information). Therefore, rather than yielding the absolute concentration of a modification in a given tissue, the data show directly the extent to which the analyzed tRNA set is modified. For representative murine and porcine tissues, an additional quantification of the ubiquitous tRNA modifications m^5^C and Ψ was performed (see Figure S4).

**Figure 2:**
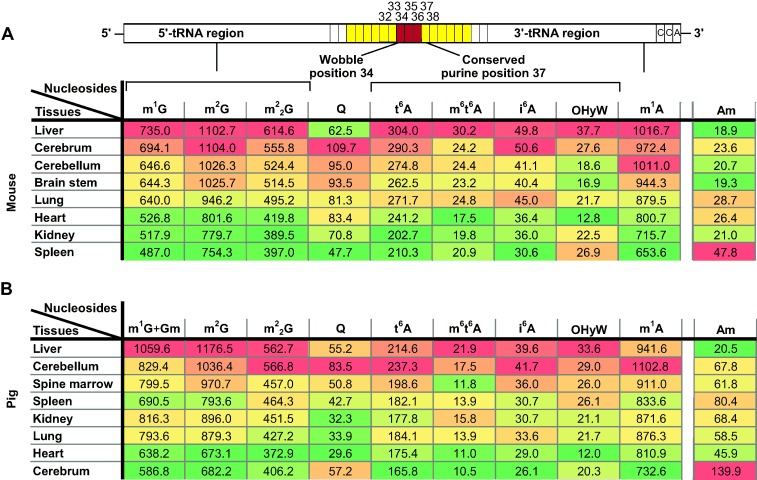
Quantitative data for the investigated tRNA modifications in various murine (A) and porcine (B) tissues. All tRNA nucleoside values are given per 1000 tRNA molecules (‰). These data reveal a similar, tissue-dependent extent of modification for all investigated modified nucleosides except Am. Color codes in (A) and (B) are based on quantile calculations; red: highest value, yellow: 50 % quantile, green: lowest value. For intermediate values appropriate intermediate shades were calculated. Despite the slight variation in the absolute quantification values, trends in modification content were conserved across different biological samples (both in mouse and pig, see Tables S2–S5 in the Supporting Information).

The data show that each tissue type incorporates different amounts of a specific modified nucleoside into the respective tRNA ensemble. While the tRNAs in liver tissue contain large numbers of modified nucleosides (colored red), those isolated from lung and kidney tissue feature far fewer modifications (colored yellow and green). It is known that the amounts of individual tRNA species vary between tissues,[11] but these variations are small in comparison to the detected changes in modification levels, arguing that tissues modify their tRNA to different extents (for a detailed discussion about the influence of codon bias see the Supporting Information). Most important in this respect is the observation that the levels of the m^1^A, m^2^G, m^5^C, and Ψ modifications (Figure 2 and Figure S4) follow the overall trend. These modifications are present in almost all tRNA species, and hence if tRNA composition would bias the quantitative data then the levels of these modifications would be expected to stay constant (or at least not follow the trend strongly).

Consistently, murine and porcine tissues show similar trends with liver, characterized by a high metabolic activity, having tRNA in both cases highly modified, while the tRNA from muscle tissue such as heart shows a rather low modification content.[12] Divergence between the two organisms is observed for some tissues such as cerebrum and spleen. This might be due to species-specific variation in tissue metabolism, or it might arise from genetic variation between species, as previously observed for bacteria.[13] Surprising is the observation that the data for Am follow a different trend, with higher Am levels found in tissues with largely unmodified tRNA (see Figure 2 and Table S8 in the Supporting Information). Because 2′-*O*-methylation stabilizes RNA,[4], [14] the observed pattern could reflect the role of this modification in stabilizing hypomodified tRNA. Furthermore, while levels of queuosine (Q) generally fit the overall trend, this nucleoside has unexpectedly high levels in brain tissue both in mouse and pig, suggesting a more complex specialized role in those tissues.[15]

In order to confirm the results, we next measured the modification content in a sequence context. To this end we carried out a parallel LC–MS analysis of partial tRNA digests (RNase A) from two representative porcine tissues, liver and heart. From the digests we obtained a number of defined tRNA fragments (small oligomers) resulting from selective cleavage after C and U. Out of the obtained fragments we determined 10 for which we were able to detect the unmodified and modified sequences using mammalian tRNA sequences from the Sprinzl tRNA database.[3b] We then determined the relative amounts of the modified versus the unmodified tRNA fragments. The extent of modification of the respective tRNA sequence was calculated directly from the ratio between the areas of the specific mass peaks for the modified and the unmodified fragments (see Figure 3 and Table S9 in the Supporting Information).[16] The results show that the representative modified nucleosides m^1^G, m^1^A, m^2^G, i^6^A, and t^6^A are indeed more abundant in tRNA fragments derived from liver, supporting the data from the isotope-dilution-based direct nucleoside quantification.

**Figure 3:**
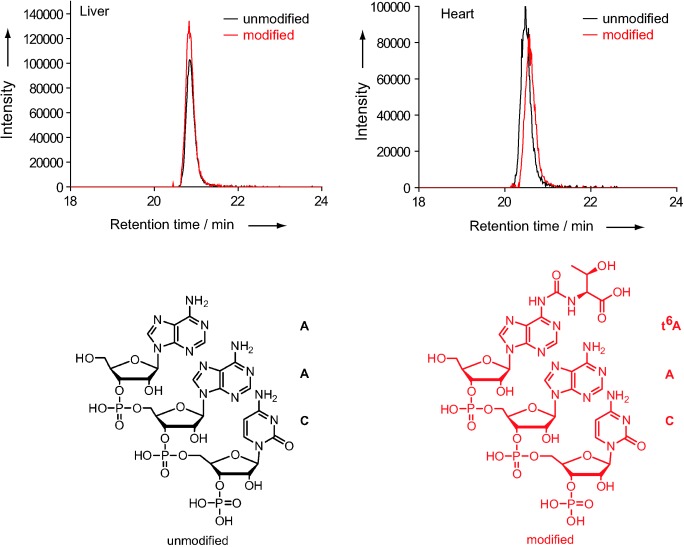
Representative qualitative comparison of amounts of unmodified RNA fragments AAC and the corresponding modified t^6^AAC in the RNase A digests of liver and heart tRNA. Overlayed LC–MS chromatograms showing ions detected at the calculated masses of the AAC (*m*/*z*=489.5682–489.5742) and t^6^AAC (*m*/*z*=562.0863–562.0933) fragments (*z*=−2) and the corresponding structures. The ratio of the peak areas of modified to unmodified fragments for liver can be seen to be higher than that for heart. Further identified fragment ratios are listed in Table S9 in the Supporting Information.

Based on the data we concluded that tissues mature their tRNA differently to satisfy individual translational needs. In support of this hypothesis we observed that published data for the rates of protein synthesis in vivo in different mammalian organs show a good level of correlation with our quantitative data for pig tissues (Figure S5 in the Supporting Information),[12, 17] suggesting that higher overall tRNA modification content might be linked to faster rates of protein translation in a certain tissue. In order to test this hypothesis directly, we analyzed the translational efficiency of tissue-specific tRNA ensembles using an in vitro coupled transcription/translation reticulocyte lysate system.[18] The original tRNAs present in the system were removed chromatographically using an ethanolamine–Sepharose column.[18] Subsequently, the tissue-extracted tRNA ensembles were added. Translational efficiency was measured by observing the increase in luminescence linked to the production of the protein luciferase (see Figure S7). The slopes of the plotted curves from at least three repeated experiments were normalized to the most efficient ensemble.

In a first set of experiments, total tRNA ensembles from porcine tissues were used. Figure 4 plots the measured rates against the corresponding normalized modification levels calculated based on LC–MS data presented in Figure 2 B (exact values are listed in Figure S8 in the Supporting Information). From Figure 4 it can be seen that the overall modification content correlates with the translational efficiency of the isolated tRNA ensemble, but the correlation is far from optimal.

**Figure 4:**
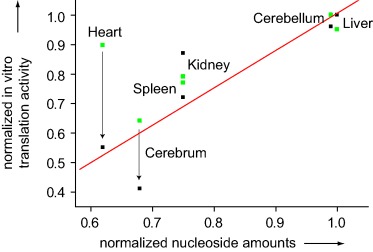
Translation activity of tRNA extracts isolated from different porcine tissues with (black squares) and without (green squares) removal of mitochondrial tRNAs. A plot of the linear fit of relative in vitro translation activity and normalized nucleoside levels shows a significant correlation after removal of mitochondria (red line; *r*=0.861, *P*=0.028).

We noted that specifically the values obtained from tissues known to be rich in mitochondria (heart in particular) deviate from the expected trend.[19] Since mitochondrial tRNA features its own set of modifications,[13] we therefore removed in a second experiment the mitochondria from the porcine tissues before the tRNA extraction (Figure S6 in the Supporting Information).[20] The obtained data for cytosolic tRNA show indeed a higher degree of correlation between the translational activity and the modification content (black squares in Figure 4; *r*=0.861, *P*=0.028), indicating that the modification level of cytosolic tRNAs is one factor that influences the efficiency of translation. This correlation was further confirmed for mouse using total tRNA ensembles extracted from tissues known to have relatively low mitochondrial tRNA content (see Figure S9). Our results are consequently in good agreement with the common idea that specific noncanonical bases fine-tune the binding of tRNAs to the ribosome. As the translation rate is determined by the competition between near-cognate and cognate aminoacyl-tRNAs,[21] a high modification level increases the affinity of the correct tRNA to the ribosome, which may allow faster discrimination.[22] This reduces the ribosome step time, which in turn may increase protein synthesis rates.

In summary, we have reported the parallel quantification of 12 modified nucleosides in tRNA ensembles from various porcine and murine tissues and showed that the overall modification content varies substantially. Furthermore, we provide evidence that the modification level correlates with the in vitro protein synthesis capacity, suggesting that the extent to which the tRNA ensemble is chemically modified modulates the translational efficiency. Our data show that the tRNA modification level is another layer of information that programs cells in terms of their translational potency.

## Supplementary material

Detailed facts of importance to specialist readers are published as ”Supporting Information”. Such documents are peer-reviewed, but not copy-edited or typeset. They are made available as submitted by the authors.
