# Supplementary material for: Isotope-Based Analysis of Modified tRNA Nucleosides Correlates Modification Density with Translational Efficiency
Source: Angew Chem Int Ed Engl. 2012 Oct 4;51(44):11162–5. doi: 10.1002/anie.201203769 (PMC3533783; doi:10.1002/anie.201203769)
Supplement: Supplementary file 1 [file anie0051-11162-SD1.pdf]

Angewandte Chemie
Eine Zeitschrift der Gesellschaft Deutscher Chemiker

Supporting Information
© Wiley-VCH 2012
69451 Weinheim, Germany

Isotope-Based Analysis of Modified tRNA Nucleosides Correlates Modification Density with Translational Efficiency**

*Caterina Brandmayr, Mirko Wagner, Tobias Brückl, Daniel Globisch, David Pearson, Andrea Christa Kneuttinger, Veronika Reiter, Antje Hienzsch, Susanne Koch, Ines Thoma, Peter Thumbs, Stylianos Michalakis, Markus Müller, Martin Biel, and Thomas Carell**

anie_201203769_sm_miscellaneous_information.pdf

Supplementary Materials and Methods

Porcine and murine tissue samples. Pork tissue was obtained from a slaughterhouse immediately after sacrifice and processed within 4 h. Prolonged waiting times were observed to result in impaired results. Heart, kidneys, liver and spleen were briefly washed with demineralized water. Cerebellum, cerebrum, spinal cord and lung were washed rigorously and superficial blood vessels were removed. Each organ was sampled at three different positions from two animals to give 6 samples in total. Tissue samples were cut out omitting surface areas. Mouse organs were prepared from C57BL/6N mice (5 weeks old) and frozen in liquid nitrogen. The organs were kept at –80°C until tRNA extraction was performed.

tRNA extraction. All extraction steps were performed on ice or at 4 °C. All extraction, desalting, and chromatography steps were performed with minor changes as described previously [1]. Pork tissue samples (5 g) or whole cerebellums, buffer 1 (15 mL, 0.01 M $Mg(OAc)_2$, 0.05 M NaOAc, 0.15 M NaCl, pH 4.5) and ice were placed into a Waring Blender. The mixture was homogenized and transferred to a 50 mL Falcon tube and extracted three times with phenol. A similar lysis protocol was used for whole mouse organs. The final purified dry tRNA pellet was dissolved in MilliQ water (200-1000 µL) for enzymatic digestion. In case the resulting tRNA concentration proved to be too low for the subsequent digestion (<140 ng/µl) another EtOH precipitation step was conducted. Purity of the tRNA samples was proven by gel electrophorsis (Fig. S10).

Enzymatic digestion. The enzymatic digestion was performed in a two step procedure as described previously [1]. All labeled nucleosides were added, followed by centrifugation of the sample (12100 g, 15 min). The supernatant was lyophilized to a total volume of 105 µL. Each pork and mouse tissue experiment was performed at least in triplicate with three independent concentrations of the appropriate labeled nucleosides. The concentrations of standard solutions were chosen to be in the expected range of the sample nucleoside concentration.

LC-ESI-MS. The samples (100 µL injection volume) were analyzed by LC-ESI-MS on a *Thermo Finnigan LTQ Orbitrap XL* and were chromatographed by a *Dionex Ultimate 3000*

HPLC system with a flow of 0.15 mL/min over an Uptisphere120-3HDO column from *Interchim*. The column temperature was maintained at 30 °C. Eluting buffers were buffer C (2 mM $HCOONH_4$ in H_2O (pH 5.5)) and buffer D (2 mM $HCOONH_4$ in H_2O/MeCN 20/80 (pH 5.5)). The gradient was 0 → 41.25 min; 0 % → 6 % buffer D; 41.25 → 80 min; 8 % → 60 % buffer D; 80 → 82 min; 60 % → 100 % buffer D; 82 → 100 min; 100 % buffer D; 100 → 105 min; 100 → 0 % buffer D; 105 → 115 min; 0 % buffer D. The elution was monitored at 260 nm (*Dionex Ultimate 3000 Diode Array Detector)*. The chromatographic eluent was directly injected into the ion source without prior splitting. Ions were scanned by use of a positive polarity mode over a full-scan range of *m/z* 200-1000 with a resolution of 30000. Parameters of the mass spectrometer were tuned with a freshly mixed solution of adenosine (5 µM) in buffer C. The parameters used in this section were sheath gas flow rate, 16 arb; auxiliary gas flow rate, 10 arb; sweep gas flow rate, 4 arb; spray voltage, 5.0 kV; capillary temperature, 200 °C; capillary voltage, 25 V, tube lens 60 V.

LC-MS of RNase A digests. RNase A digests were performed by incubation of tRNA (10 µg) with RNase A (1 or 10 µg, Fermentas) in 100 mM ammonium acetate buffer at a total volume of 100 µL at 37 ºC for 2 h. Whole digested samples were analyzed by the same LC-ESI-MS system as for the quantitative analysis with a flow of 0.15 mL/min (*Thermo Finnigan LTQ Orbitrap XL*; *Dionex Ultimate 3000 HPLC* system; Uptisphere120-3HDO column from *Interchim*). The column temperature was maintained at 30 °C. We used the same eluting buffers C and D. The gradient was 0 min → 10 min; 0 % buffer D; 10 → 20 min; 0 % → 10 % buffer D; 20 min → 42 min; 10 % → 40 % buffer D; 42 min → 50 min; 40 % → 100 %; 50 min → 60 min; 100 % buffer D; 60 min → 70 min; 100 % → 0 % buffer D; 70 min → 80 min; 0 % buffer D. The parameters used in this section were sheath gas flow rate, 30 arb; auxiliary gas flow rate, 10 arb; sweep gas flow rate, 4 arb; spray voltage, 2.3 kV; capillary temperature, 200 °C; capillary voltage, -20 V, tube lens -93 V. Ions were scanned by use of a negative polarity mode over a full-scan range of *m/z* 200-1500 with a resolution of 30000. Parameters of the mass spectrometer were tuned with a solution of the RNA 7-mer AUUCCCG (5 µM) in buffer C.

Separation of mitochondria and cytosol. Isolation of mitochondria from porcine organs was done by fractionated centrifugation at low and high speed according to the Mitochondria Isolation Kit from Sigma-Aldrich.[2]

Cytochrome C oxidase assay. The respiratory activity of mitochondria in whole cell lysates and in enriched fractions of pork tissue was measured using the cytochrome C oxidase Assay Kit of Sigma-Aldrich.[3]

***In vitro* translation assay.** For *in vitro* translation activity measurements we used the combined transcription and translation reticulocyte assay kit from Promega (TNT Coupled Reticulocyte Lysate System). tRNAs present in the reticulocyte lysate were removed by chromatography using an ethyanolamine-Sepharose column according a previous report [4]. The chromatographic step was performed at 4 °C and collected fractions were frozen at -80 °C. Briefly, we supplemented the lysate with 20 µM hemin, 50 mM KCl, and 0.5 mM $MgCl_2$ prior to chromatography. The column was equilibrated with buffer H (75 mM KCl, 1.6 mM $MgCl_2$, 10 mM NaCl, 0.1 mM EDTA, 1 mM DTT, 10 mM HEPES pH = 7.2). The supplemented extract (700 µL) was loaded on the column and eluted with 1.0 mL of buffer H. Fractions of 50 µL were collected and assayed for tRNA dependent translational activity before usage. The column was regenerated between different filtration steps with buffer I (500 mM KCl, 1,6 mM $MgCl_2$, 10 mM NaCl, 0,1 mM EDTA, 1 mM DTT, 10 mM HEPES pH = 7.2) and stored at 4 °C. The collected tRNA-depleted fractions were used for *in vitro* translation experiments and stored at -80 °C.

For the *in vitro* translation assay, we used the non-radioactive luciferase control reaction with TNT RNA Polymerase T7 and Luciferase control DNA as described in the Promega Kit with minor changes. Each assay contained TNT reaction buffer (0.5 µL), T7 TNT RNA Polymerase (0.25 µL), amino acid mixture minus leucine (1 mM), amino acid mixture minus methionine (1mM), RNasin ribonuclease inhibitor (10 U), Luciferase T7 control DNA (0.25 µg). Afterwards the appropriate tRNA (12.5 ng) and RNase-free water were added to a total assay volume of 6.25 µL, followed by addition of tRNA-depleted lysate (6.25 µL).

These translation reactions were incubated at 30 °C and a 1 µL aliquot was removed every 2-3 min from each fraction starting at 12 min. This sample was transferred into a 96 well plate, mixed well with 25 µL of Luciferase Assay substrate and analyzed immediately.

Luminescence was measured with a TECAN Microplate Reader Genios Pro in 10 cycles for 100 ms each cycle. Reproducible luminescence slopes were obtained after normalization to the liver value and the results are shown in Fig S7. The data were averaged and plotted against time. A linear fit of each initial slope was performed and normalized to the highest value (usually liver). Every measurement was repeated at least in triplicate and every value represents mean value with s.d. Care was taken to use different tRNA-depleted fractions.

Calibration curves. Mass calibration curves of the labeled and corresponding unlabeled synthesized nucleosides were obtained at five different concentration ratios. For each concentration an average value of three independent measurements was determined (Fig. S2). Each labeled nucleoside solution was mixed with three different concentrations of the corresponding unlabeled nucleosides. The areas of labeled and unlabeled nucleosides from LC-MS measurements were determined using the *Qualbrowser* program by extraction of the accurate mass range with a mass filter (Table S1) from the total ion current (TIC). The linear fits of the determined area ratios with the amount ratios gave R^2-values of minimum 0.9992. The linear fit equations were used for calculation of the exact nucleoside contents in bulk tRNA samples. Synthetic labeled nucleosides were added to the digest solutions and the areas of labeled and unlabeled nucleosides were determined as described above. The amount of each nucleoside (Table S2-7) was calculated from the obtained area ratios and the linear fit equations of the calibration curves.

Ψ calibration curves (see Fig. S3) were obtained by addition of three different known amounts of non labeled Ψ standard to three of four liver tRNA extract samples previously digested as described above. The samples were measured by HPLC-ESI-MS together with the non-spiked liver tRNA extract sample. The ion current area of the non-spiked sample was subtracted from the ion current areas of the spiked samples and the resulting values were plotted against the amount of Ψ added. The derived linear fit equation was used for quantification of Ψ in the investigated samples.

Intra- and Inter-assay tests. The intra-assay test was performed for representative nucleosides m^1A, i^6A, ms^2i^6A, and m^1G. The determined values of labeled to unlabeled nucleosides using the calibration curves of a sample after enzymatic digestion showed good reproducibility for each nucleoside (N=5); 2.5% for m^1A, 0.4% for i^6A, 0.7% for ms^2i^6A, and

2.6% for m^1G. The single area values of each labeled or unlabeled nucleoside in the intra-assay test resulted in an average value of 4.1%. The inter-assay test gave an area ratio reproducibility (N=6) of 6.3% for m^1A, 1.0% for i^6A, 1.8% for ms^2i^6A, and 4.3% for m^1G on six subsequent days. The determined single area values for each nucleoside resulted in strong variations and large average error of 34.5%. Using the area ratio with the calibration curves we gained perfect reproducibility (Table S10). No memory effect was observed during blank LC/MS experiments performed after several measurements of a sample. The blank analyses were not contaminated by carry-over.

Quantification of the Ψ nucleoside in murine and porcine tissues. Biological samples from different murine and porcine tissues were analyzed by HPLC-ESI-MS without addition of standards and quantification of Ψ was obtained from the ion current area of the unlabeled Ψ in each sample (accurate mass range is given in Table S1) and the linear fit equation of the calibration curve (see Fig.S3). The HPLC-ESI-MS protocol used was similar to that used for quantification by isotope-dilution (see above). Care was taken to perform all the measurements and the data collection for the calibration curve during the same day so to avoid variations in the mass detection accuracy.

Influence of codon bias on the *in vitro* translation activity experiment. Observed differences in translation activity could be attributed both to a variable composition of isoacceptor tRNAs and differences in tRNA modification levels. A tissue-specific codon bias has in fact been statistically proven in mammals by Plotkin *et al.*,[5] but they also show that the same tissues of evolutionary closely related species feature the same codon usage. From our analysis of the tRNA ensembles of cerebrum and spleen we can deduce that in our experiments codon bias only plays a minor role. As shown in our data, the porcine spleen tRNA set shows a higher level of modification than the cerebrum tRNAs. This is in good accordance with our observation that the *in vitro* translation activity of porcine spleen tRNA is higher than that of cerebrum. In the case of mouse we observe the opposite behavior. The murine spleen tRNA set is less modified than its cerebrum tRNAs, which goes in line with a lower translation activity of murine spleen tRNA *in vitro* compared to cerebrum.

If codon usage strongly biased the *in vitro* translation results presented here, we would expect similar outcomes for both organisms regarding the translational activity in a certain tissue. This is not the case.

Supplementary Figures

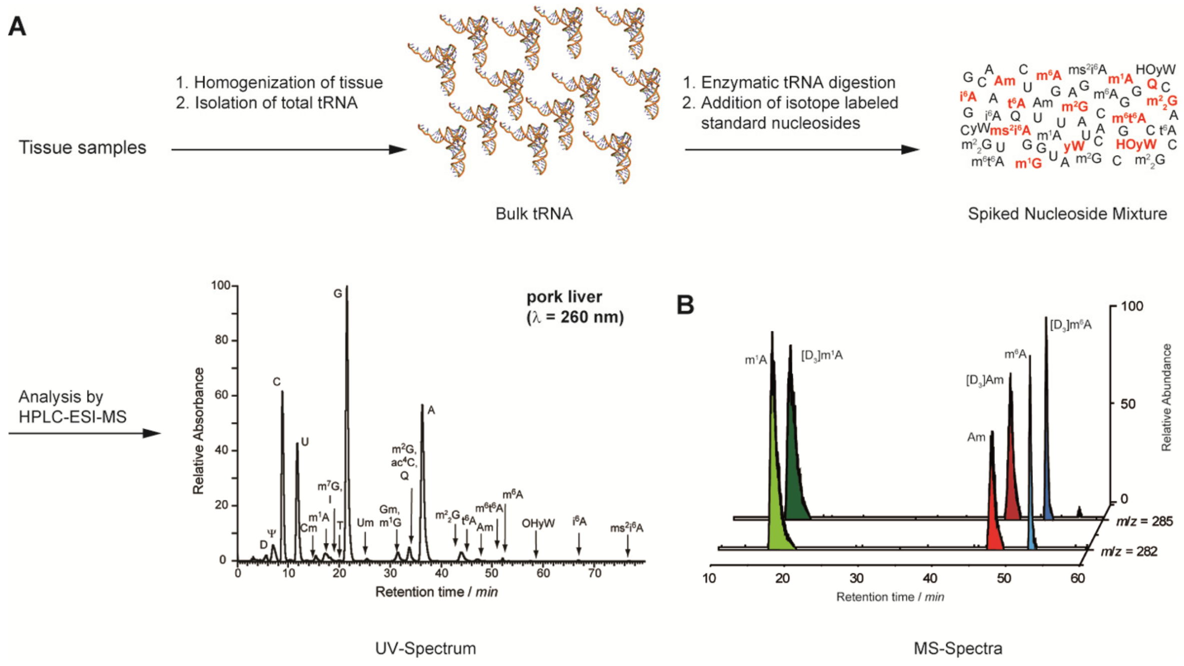

Fig. S1. Illustration of the isotope dilution method used to quantify modified tRNA nucleosides. A) The general method used, starting with homogenization of different tissue samples and ending with LC-MS analysis. The red colour letters represent the added isotope-labeled derivatives. B) Example of the two ion currents obtained for the natural monomethylated adenosines m^{1}A, Am and m^{6}A and the corresponding labeled nucleosides d_3-m^{1}A, d_3-Am and d_3-m^{6}A.

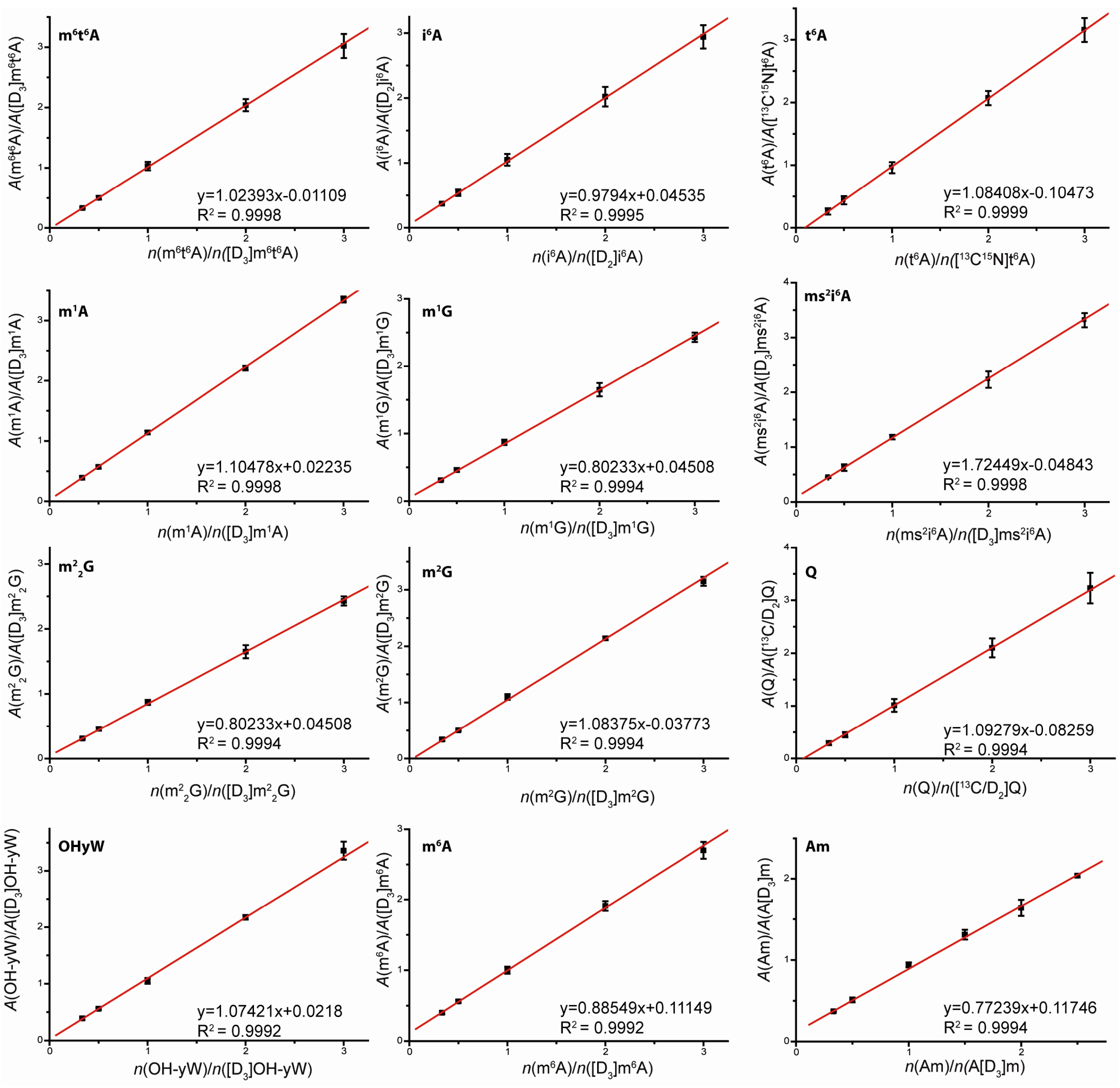

Fig. S2. Calibration curves for the nucleosides synthesized and used for quantification: m^6t^6A, i^6A, t^6A, m^1A, m^1G, ms^2i^6A, $m^2{}_2G$, m^2G, Q, OHyW, m^6A, and Am, with an average R^2 value of 0.9995. Quantitative data for the modification m^6A are not presented due to rearrangement from m^1A to m^6A. Inter-and intra-assays proved constant values of m^1A and varying values of m^6A. The modifications m^1G and Gm were quantified with d_3-m^1G because of overlapping UV and mass signals.

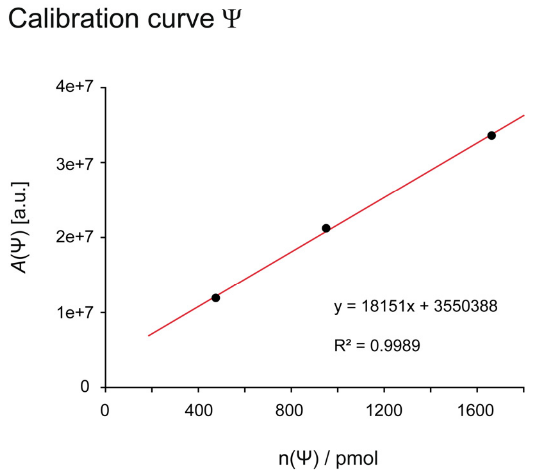

Fig. S3. Calibration curve for the tRNA nucleoside Ψ with a R^2 value of 0.9989 (for details, see above). Care was taken to perform data collection for the calibration curve and the measurements of the biological samples during the same day so to avoid variations in the mass detection accuracy.

A — Mouse

Tissues \ Nucleosides	m⁵C	Ψ
Liver	1888.0	1986.9
Cerebrum	1831.5	1639.2
Lung	1747.8	1436.9
Kidney	1316.5	924.5
Spleen	1368.5	1284.7

B — Pork

Tissues \ Nucleosides	m¹G	m⁵C	Ψ
Liver	746.7	2742.5	2263.1
Spleen	627.1	2687.5	2146.1
Kidney	628.8	2764.5	1886.8
Lung	575.8	2452.7	1907.6
Heart	535.1	2138.1	1776.1
Cerebrum	448.5	1936.4	1330.4

Fig. S4. Quantitative data for the tRNA nucleosides m^1G, m^5C and Ψ in various representative murine (*A*) and porcine (*B*) tissues. All tRNA nucleoside values are given per 1000 tRNA molecules (‰). These data reveal a similar, tissue-dependent extent of modification for the investigated modified nucleosides, corresponding to the trend shown in the main paper. The Ψ values were determined by the method described above without addition of an isotope-labeled derivative. A separate quantification of m^1G for porcine tissues (*B*) was possible using the optimized LC-method. Color codes in (*A*) and (*B*) are based on quantile calculations; red: highest value, yellow: 50% quantile, green: lowest value. For intermediate values appropriate shades of color were calculated, standard deviations are listed in Tables S6-S7.

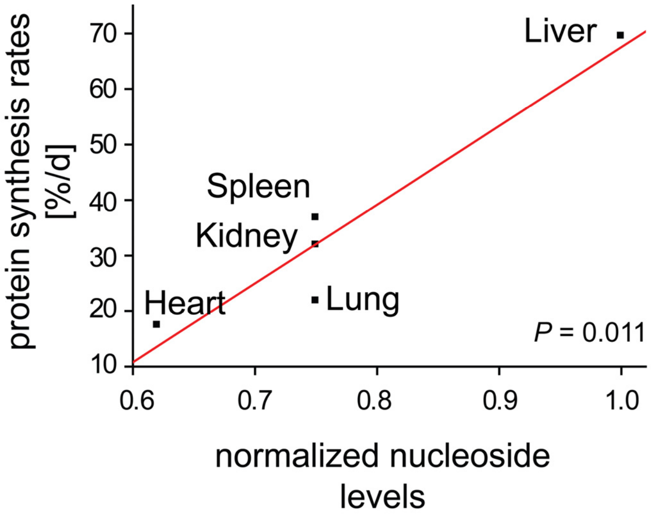

Fig. S5. Correlation of *in vivo* protein synthesis rates with normalized nucleoside levels. This correlation shows a high significance with $P = 0.011$ [6].

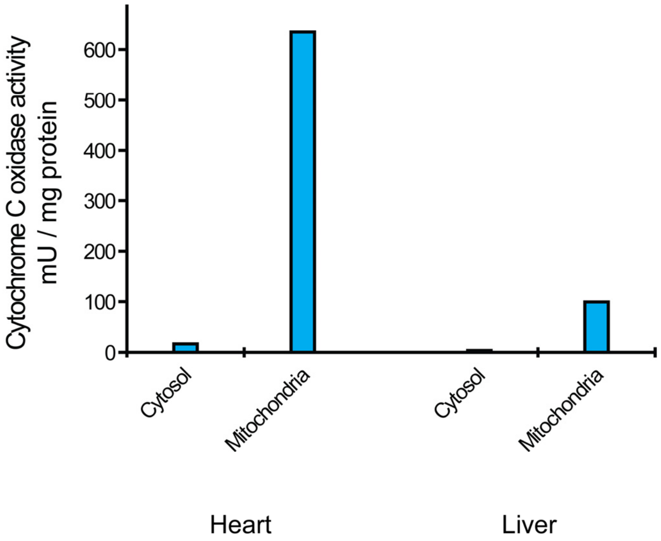

Fig. S6. Cytochrome C oxidase activity after separation of mitochondria and cytosol in porcine tissues. Activity of cytochrome C oxidase was taken as a measure of the mitochondrial content in each fraction. Values for the activity are increased in mitochondrial fractions and decreased in cytosolic fractions for the two representative tissues heart and liver, therefore providing evidence for an enrichment of mitochondria in mitochondrial fractions and suggesting an almost mitochondria-free cytosolic fraction.

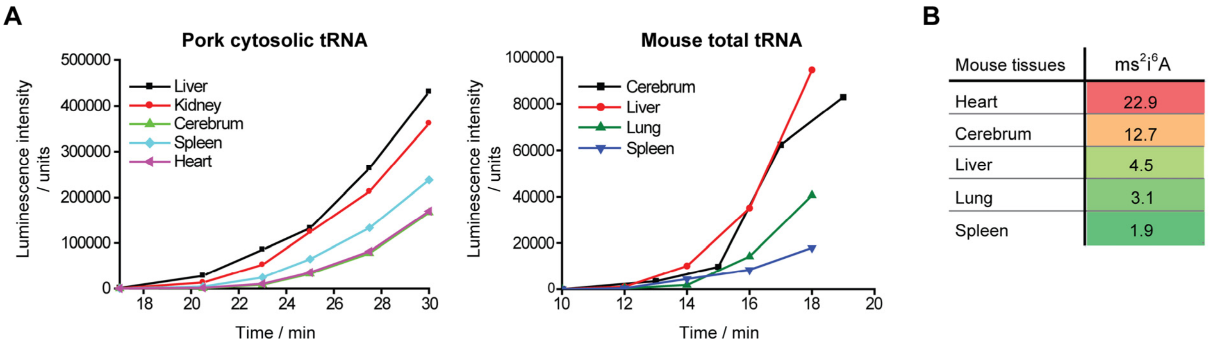

Fig. S7. (A) Representative *in vitro* translation experiment read out of pork cytosolic tRNAs (liver, kidney, cerebrum, spleen, heart) and mouse total tRNAs (cerebrum, liver, lung and spleen). Mouse tissues were selected based on low mitochondrial content, as shown by the relatively low content of ms^2i^6A modification (see (B)), which is known to only occur in mitochondrial tRNA.[3] Heart is listed to provide a comparison with a tissue known to contain a high proportion of mitochondrial tRNA. This experiment was performed at least in triplicate. Initial rates were calculated in the range of 17-25 min and normalized to liver in the case of pork samples. For mouse tRNA ensembles, rates were calculated from data points between 12-16 minutes and normalized to cerebrum.

Tissue	normalized nucleoside levels[a]	normalized *in vitro* translation activity (total tRNA)	normalized *in vitro* translation activity (cytosolic tRNA)
Liver	1.00	0.95 ± 0.02	1.00
Cerebellum	0.99 ± 0.23	1.00	0.96 ± 0.05
Spleen	0.75 ± 0.08	0.77 ± 0.22	0.72 ± 0.12
Kidney	0.75 ± 0.09	0.79 ± 0.07	0.87 ± 0.01
Cerebrum	0.68 ± 0.17	0.64 ± 0.02	0.41 ± 0.04
Heart	0.62 ± 0.14	0.90 ± 0.04	0.55 ± 0.14

Fig. S8. Translational activity of tRNA sets from pork tissues. Average normalized nucleoside levels of pork liver, cerebellum, spleen, kidney, cerebrum, and heart (calculated from LC-MS data presented in Fig. 2*B*) and relative *in vitro* translation activities of cytosolic and total tRNA. All values are normalized to the highest value. Standard deviations (mean ± s.d.) are given for the other tissues. Note: while the error values here are relatively large, these represent the variation over all modified nucleosides. The measurements for each nucleoside have low errors (~5%), and they show the same relationship as the averaged set.

A

Tissue	normalized nucleoside levels	normalized *in vitro* translation activity (total tRNA)		
		replicate 1	replicate 2	replicate 3
Liver	1.00	0.42 ± 0.05	0.58 ± 0.23	0.72 ± 0.19
Cerebrum	0.969 ± 0.05	1.00	0.39 ± 0.17	-
Lung	0.855 ± 0.06	0.19 ± 0.09	0.21 ± 0.12	0.30 ± 0.10
Spleen	0.675 ± 0.09	0.01 ± 0.001	0.08 ± 0.00	0.31 ± 0.002

B

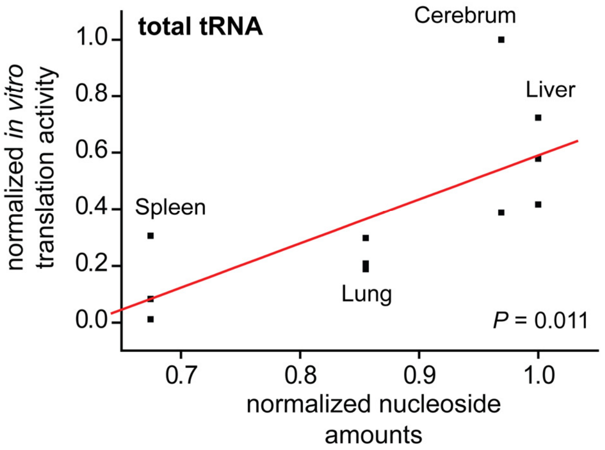

Fig. S9. Translational activity of tRNA sets from mouse tissues. (A) Average normalized nucleoside levels of mouse liver, cerebrum, lung and spleen (calculated from LC-MS data presented in Fig. 2*A*) and corresponding *in vitro* translation rates for each replicate. (B) Linear fit of normalized in vitro translational acitivity of total murine tRNAs and normalized nucleoside levels showing a considerable correlation (r = 0.723, $P = 0.011$). Each data set was normalized to its corresponding highest value.

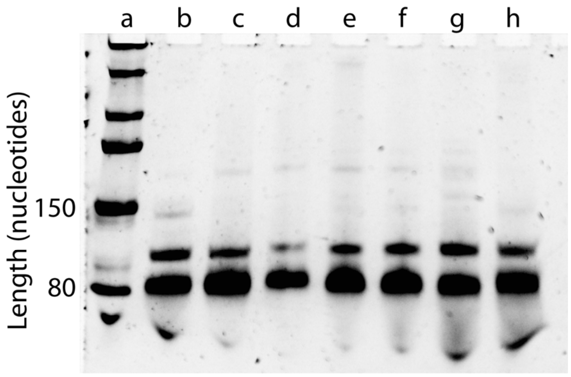

Fig. S10. RNA PAGE gel for mouse tRNA extracts from different tissues. a) Marker, b) Liver, c) Cerebrum, d) Cerebellum, e) Brain stem, f) Lung, g) Spleen, h) Kidney. As previously reported by others, there is a small but constant contamination of 5S rRNA. This contamination is observed for all tissues and therefore does not bias the results reported in this publication [7].

Supplementary Tables

Nucleoside	Nucleosides mass range *m/z*	Labeled nucleosides mass range *m/z*
Am, m^1A	282.1142-282.1262	285.1335-285.1435
t^6A	413.1315-413.1475	418.1420-418.1580
i^6A	336.1606-336.1716	338.1740-338.1840
ms^2i^6A	382.1484-382.1594	385.1679-385.1789
$m^2{}_2G$	312.1248-312.1368	315.1441-315.1561
m^2G, m^1G, Gm	298.1076-298.1196	301.1276-301.1396
Q	410.1640-410.1730	413.1778-413.1898
m^6t^6A	427.1532-427.1622	430.1707-430.1807
OHyW	525.1879-525.2029	528.2065-528.2195
m^5C	258.1034-258.1134	261.1223-261.1323
Ψ	245.0708-245.0828	-

Table S1. High resolution mass ranges of natural and corresponding labeled nucleosides used for quantification. Modifications 3'-adjacent to the anticodon loop: hydroxywybutosine (OHyW), N^6-isopentenyladenosine (i^6A), N^6-methyl-N^6-threonylcarbamoyladenosine (m^6t^6A), N^6-threonylcarbamoyladenosine (t^6A), and 1-methylguanosine (m^1G). Modifications in the wobble position: queuosine (Q) and 2'-*O*-methylguanosine (Gm). Modifications in other positions: 1-methyladenosine (m^1A), 2'-*O*-methyladenosine (Am), m^1G, N^2-methylguanosine (m^2G), N^2,N^2-dimethylguanosine ($m^2{}_2G$), 5-methylcytosine (m^5C), pseudouridine (Ψ) and Gm.

Mouse Tissues		Am	t^6A	i^6A	$m^2{}_2G$	m^2G	Q	OHyW	m^1G	m^6t^6A	m^1A
**Kidney**	Mean value	21.0	202.7	36.0	389.5	779.7	70.8	22.5	517.9	19.8	715.7
	Standard deviation	3.6	5.8	0.9	12.3	40.3	6.2	4.3	23.0	1.3	66.0
	Standard deviation in %	17.2	2.9	2.5	3.2	5.2	8.8	19.3	4.4	6.7	9.2
**Lung**	Mean value	28.7	271.7	45.0	495.2	946.2	81.3	21.7	640.0	24.8	876.5
	Standard deviation	18.2	18.3	1.9	30.5	48.1	7.4	3.4	21.5	1.8	54.2
	Standard deviation in %	63.2	6.7	4.3	6.2	5.1	9.2	15.9	3.4	7.3	6.2
**Spleen**	Mean value	47.8	210.3	30.6	397.0	754.3	47.7	26.9	487.0	20.9	653.6
	Standard deviation	1.4	7.8	2.1	3.2	43.9	17.4	11.4	26.3	0.9	60.1
	Standard deviation in %	2.9	3.7	6.8	0.8	5.8	36.5	42.4	5.4	4.3	9.2
**Liver**	Mean value	18.9	304.0	49.8	614.6	1102.7	62.5	37.7	735.0	30.2	1016.7
	Standard deviation	9.6	7.5	1.0	14.2	34.8	7.7	9.1	14.2	0.7	150.2
	Standard deviation in %	50.7	2.5	2.1	2.3	3.2	12.3	24.3	1.9	2.3	14.8
**Cerebrum**	Mean value	23.6	290.3	50.6	555.8	1104.0	109.7	27.6	694.1	24.2	972.4
	Standard deviation	1.7	10.8	3.0	23.0	33.8	11.9	2.4	24.2	0.6	39.9
	Standard deviation in %	7.4	3.7	5.9	4.1	3.1	10.9	8.6	3.5	2.5	4.1
**Cerebellum**	Mean value	20.7	274.8	41.1	524.4	1026.3	95.0	18.6	646.6	24.4	1011.0
	Standard deviation	1.4	14.9	1.7	18.1	67.7	9.1	3.3	35.3	0.9	118.9
	Standard deviation in %	6.6	5.4	4.2	3.5	6.6	9.6	17.6	5.5	3.5	11.8
**Heart**	Mean value	26.4	241.2	36.4	419.8	801.6	83.4	12.8	526.8	17.5	800.7
	Standard deviation	9.5	26.6	5.3	44.9	108.1	13.6	2.7	61.1	2.7	113.2
	Standard deviation in %	35.9	11.0	14.4	10.7	13.5	16.3	20.8	11.6	15.4	14.1

Mouse Tissues		Am	t^6A	i^6A	m^2_2G	m^2G	Q	OHyW	m^1G	m^6t^6A	m^1A
**Brain stem**	Mean value	19.3	262.5	40.4	514.5	1025.7	93.5	16.9	644.3	23.2	944.3
	Standard deviation	3.6	9.5	2.7	23.4	72.1	6.7	3.4	39.2	0.7	111.0
	Standard deviation in %	18.7	3.6	6.6	4.5	7.0	7.2	20.5	6.1	3.1	11.8

Table S2. Modification numbers per 1000 tRNAs in different murine tissues. The table lists the average values calculated from the modification content of two sets of five animals. For each sample at least three independent digests and measurements were performed. 10,3 % mean standard deviation was obtained for all nucleosides excluding Am. 22,3 % mean standard deviation was obtained for Am. As noted in the Table S3, the overall mean standard deviation across nucleosides and tissues for the two individual sample sets is below 5%.

Mouse Tissues		Am	t^6A	i^6A	m^2_2G	m^2G	Q	OHyW	m^1G	m^6t^6A	m^1A
**Lung A**	Mean value	49.6	255.8	43.0	479.9	938.6	90.1	23.9	634.8	24.4	852.3
	Standard deviation	0.6	15.5	0.8	27.9	62.0	1.2	3.1	30.3	2.0	59.1
	Standard deviation in %	1.3	6.1	1.8	5.8	6.6	1.3	13.0	4.8	8.2	6.9
**Lung B**	Mean value	13.1	283.6	46.5	506.6	951.9	75.3	20.0	643.9	25.2	900.0
	Standard deviation	2.1	8.5	0.9	27.2	33.0	2.0	2.6	9.1	1.6	39.4
	Standard deviation in %	16.1	3.0	1.9	5.4	3.5	2.6	13.0	1.4	6.2	4.4
**Spleen A**	Mean value	47.2	217.4	32.2	397.8	797.7	67.7	37.7	508.6	21.5	646.3
	Standard deviation	0.2	4.7	0.4	3.7	8.5	0.7	2.2	18.7	0.8	30.2
	Standard deviation in %	0.4	2.1	1.4	0.9	1.1	1.0	5.7	3.7	3.5	4.7
**Spleen B**	Mean value	48.3	203.2	29.1	396.5	711.0	32.7	16.1	465.5	20.3	660.9
	Standard deviation	1.8	0.9	1.9	2.6	6.0	1.7	4.8	10.4	0.6	78.7
	Standard deviation in %	3.7	0.5	6.6	0.7	0.8	5.2	29.7	2.2	3.0	11.9
**Liver A**	Mean value	28.4	310.6	50.1	612.6	1107.8	68.8	45.7	744.1	30.4	895.9
	Standard deviation	0.7	4.4	0.4	3.2	39.5	0.7	2.0	13.6	0.9	9.1
	Standard deviation in %	2.5	1.4	0.8	0.5	3.6	1.0	4.3	1.8	2.9	1.0
**Liver B**	Mean value	9.4	297.4	49.5	616.5	1097.7	54.2	29.7	725.8	30.0	1137.6
	Standard deviation	1.9	2.7	1.4	19.7	28.6	3.7	5.9	7.1	0.3	125.8
	Standard deviation in %	20.0	0.9	2.7	3.2	2.6	6.8	20.0	1.0	0.8	11.1
**Cerebrum A**	Mean value	22.2	298.6	52.8	572.2	1131.5	113.8	28.8	713.0	24.3	963.2
	Standard deviation	0.9	5.0	1.2	8.4	15.3	4.8	1.7	9.3	0.7	46.4
	Standard deviation in %	3.9	1.7	2.2	1.5	1.4	4.3	6.0	1.3	2.7	4.8

Mouse Tissues		Am	t^6A	i^6A	$m^2{}_2G$	m^2G	Q	OHyW	m^1G	m^6t^6A	m^1A
**Cerebrum B**	Mean value	25.3	279.2	47.7	533.9	1067.3	104.2	25.2	668.9	23.9	984.6
	Standard deviation	0.7	5.2	1.8	17.4	2.7	15.8	1.4	11.6	0.4	24.2
	Standard deviation in %	2.9	1.8	3.9	3.3	0.2	15.1	5.7	1.7	1.6	2.5
**Cerebellum A**	Mean value	19.3	291.2	42.8	544.7	1096.1	102.7	20.7	684.4	25.2	1082.0
	Standard deviation	0.6	6.3	0.5	5.1	23.3	6.6	2.8	19.5	0.4	129.9
	Standard deviation in %	3.0	2.2	1.3	0.9	2.1	6.4	13.3	2.9	1.8	12.0
**Cerebellum B**	Mean value	21.8	262.6	39.9	509.2	974.0	87.3	17.0	618.2	23.8	957.8
	Standard deviation	0.7	3.0	1.2	3.8	35.1	2.2	2.6	3.8	0.5	74.0
	Standard deviation in %	3.1	1.1	3.0	0.8	3.6	2.5	15.5	0.6	2.0	7.7
**Heart A**	Mean value	17.0	266.2	41.1	461.9	905.8	93.9	15.7	584.3	20.1	891.9
	Standard deviation	0.6	8.0	2.4	12.8	30.2	7.7	0.3	21.7	0.9	82.1
	Standard deviation in %	3.5	3.0	5.8	2.8	3.3	8.2	1.7	3.7	4.4	9.2
**Heart B**	Mean value	35.7	216.3	31.6	377.6	697.4	69.4	10.7	469.4	14.9	709.4
	Standard deviation	2.3	10.4	2.0	17.8	27.1	3.4	1.2	20.1	0.6	47.1
	Standard deviation in %	6.6	4.8	6.3	4.7	3.9	4.9	11.7	4.3	3.7	6.6
**Brain stem A**	Mean value	14.5	274.8	43.5	543.1	1091.9	87.0	20.9	692.7	23.8	1023.2
	Standard deviation	1.2	3.6	1.5	0.4	32.1	-	1.0	3.3	0.9	40.7
	Standard deviation in %	8.6	1.3	3.4	0.1	2.9	-	5.0	0.5	3.6	4.0
**Brain stem B**	Mean value	21.7	256.4	38.9	500.2	992.5	95.1	14.8	620.2	22.8	904.8
	Standard deviation	1.3	4.0	1.6	14.3	63.2	6.6	2.2	23.4	0.4	113.9
	Standard deviation in %	5.9	1.6	4.2	2.8	6.4	6.9	14.8	3.8	1.5	12.6

Mouse Tissues		Am	t^6A	i^6A	m^2_2G	m^2G	Q	OHyW	m^1G	m^6t^6A	m^1A
**Kidney A**	Mean value	24.6	204.2	36.4	376.3	810.6	74.4	25.9	540.1	20.7	770.0
	Standard deviation	0.6	7.6	0.6	2.9	26.6	4.5	0.5	5.0	0.9	9.8
	Standard deviation in %	2.3	3.7	1.7	0.8	3.3	6.0	2.1	0.9	4.4	1.3
**Kidney B**	Mean value	17.5	201.1	35.6	399.4	748.8	66.3	19.1	495.7	18.6	675.0
	Standard deviation	0.9	2.4	1.0	5.5	25.0	5.3	3.8	6.3	0.6	60.6
	Standard deviation in %	5.2	1.2	2.7	1.4	3.3	7.9	19.8	1.3	3.2	9.0

Table S3. Modification numbers per 1000 tRNAs in different murine tissues. The table lists the average values calculated from the modification content of each individual set of five animals. For each sample at least three independent digests and measurements were performed. 4,8 % mean standard deviation was obtained for all nucleosides excluding Am. 5,0 % mean standard deviation was obtained for Am.

Pork Tissues		Am	t^6A	i^6A	m^2_2G	m^2G	Q	OHyW	m^1G+Gm	m^6t^6A	m^1A
**Heart**	Mean value	45.9	175.4	29.0	372.9	673.1	29.6	12.0	638.2	11.0	810.9
	Standard deviation	3.3	8.2	2.7	22.7	61.3	0.0	0.0	3.0	0.4	19.1
	Standard deviation in %	7.2	4.7	9.4	6.1	9.1	0.0	0.3	0.5	3.5	2.4
**Liver**	Mean value	20.5	214.6	39.6	562.7	1,176.5	55.2	33.6	1,059.6	21.9	941.6
	Standard deviation	4.8	6.4	2.7	26.1	54.7	4.6	2.9	43.6	1.8	23.6
	Standard deviation in %	23.2	3.0	6.8	4.6	4.7	8.4	8.7	4.1	8.0	2.5
**Kidney**	Mean value	68.4	177.8	30.7	451.5	896.0	32.3	21.1	816.3	15.8	871.6
	Standard deviation	19.1	7.7	0.5	25.4	55.0	13.8	4.7	40.3	1.5	35.5
	Standard deviation in %	28.0	4.3	1.8	5.6	6.1	42.8	22.3	4.9	9.5	4.1
**Spleen**	Mean value	80.4	182.1	30.7	464.3	793.6	42.7	26.1	690.5	13.9	833.6
	Standard deviation	12.6	8.9	1.0	19.6	29.5	5.9	2.2	13.4	0.4	45.2
	Standard deviation in %	15.6	4.9	3.2	4.2	3.7	13.8	8.4	1.9	2.9	5.4
**Lung**	Mean value	58.5	184.1	33.6	427.2	879.3	33.9	21.7	793.6	13.9	876.3
	Standard deviation	16.3	8.5	2.9	27.0	45.4	11.3	1.5	28.2	1.1	16.1
	Standard deviation in %	27.8	4.6	8.5	6.3	5.2	33.3	7.0	3.6	7.9	1.8
**Spine marrow**	Mean value	61.8	198.6	36.0	457.0	970.7	50.8	26.0	799.5	11.8	911.0
	Standard deviation	1.7	2.7	0.1	2.8	13.1	0.3	0.1	1.5	0.1	11.4
	Standard deviation in %	2.7	1.4	0.3	0.6	1.3	0.6	0.3	0.2	1.0	1.2
**Cerebellum**	Mean value	67.8	237.3	41.7	566.8	1,036.4	83.5	29.0	829.4	17.5	1,102.8
	Standard deviation	6.1	0.6	0.3	1.2	1.2	4.8	1.9	13.8	0.0	6.7
	Standard deviation in %	9.0	0.2	0.6	0.2	0.1	5.7	6.5	1.7	0.2	0.6

Pork Tissues		Am	t^6A	i^6A	m^2_2G	m^2G	Q	OHyW	m^1G+Gm	m^6t^6A	m^1A
**Cerebrum**	Mean value	139.9	165.8	26.1	406.2	682.2	57.2	20.3	586.8	10.5	732.6
	Standard deviation	15.9	2.8	1.5	7.3	34.6	3.8	1.2	0.1	0.6	26.3
	Standard deviation in %	11.3	1.7	5.9	1.8	5.1	6.6	5.7	0.0	5.9	3.6

Table S4. Modification numbers per 1000 tRNAs in different porcine tissues. The table lists the average values calculated from the modification content of two different animals. From each animal at least two independent samples were investigated. For each sample at least three independent digests and measurements were performed. 5.9 % mean standard deviation was obtained for all nucleosides excluding Am. 14.7 % mean standard deviation was obtained for Am.

Pork Tissues		Am	t^6A	i^6A	m^2_2G	m^2G	Q	OHyW	m^1G+Gm	m^6t^6A	m^1A
**Heart 1**	Mean value	48.2	169.6	30.9	356.8	629.8	29.6	12.0	636.0	11.2	797.4
	Standard deviation	9.7	6.1	1.1	4.0	18.3	1.1	0.5	34.6	0.5	10.4
	Standard deviation in %	20.1	3.6	3.7	1.1	2.9	3.9	3.8	5.4	4.2	1.3
**Heart 2**	Mean value	43.5	181.3	27.1	389.0	716.5	29.6	12.1	640.3	10.7	824.5
	Standard deviation	12.1	7.1	3.0	40.7	85.2	0.9	1.1	38.8	1.1	28.5
	Standard deviation in %	27.8	3.9	11.0	10.5	11.9	3.1	9.0	6.1	10.5	3.5
**Liver 1**	Mean value	23.8	210.1	37.7	544.2	1,137.8	58.5	35.7	1,028.7	20.6	958.3
	Standard deviation	2.2	2.2	0.4	18.1	0.8	4.6	1.6	7.7	0.8	39.4
	Standard deviation in %	9.4	1.0	1.1	3.3	0.1	7.9	4.6	0.8	3.7	4.1
**Liver 2**	Mean value	17.1	219.1	41.5	581.1	1,215.2	51.9	31.5	1,090.4	23.1	925.0
	Standard deviation	1.2	12.1	1.9	39.5	86.9	1.9	3.4	39.5	0.9	30.0
	Standard deviation in %	6.8	5.5	4.6	6.8	7.2	3.7	10.9	3.6	3.9	3.2
**Kidney 1**	Mean value	81.9	172.4	30.3	433.5	857.1	22.5	17.8	787.9	14.8	846.5
	Standard deviation	23.7	19.1	2.8	30.1	89.1	2.1	2.1	43.0	2.0	25.5
	Standard deviation in %	28.9	11.1	9.4	6.9	10.4	9.4	11.6	5.5	13.3	3.0
**Kidney 2**	Mean value	54.8	183.3	31.1	469.5	934.9	42.1	24.5	844.8	16.9	896.7
	Standard deviation	15.8	2.9	2.9	3.7	10.7	0.1	3.8	3.8	0.4	40.4
	Standard deviation in %	28.8	1.6	9.2	0.8	1.1	0.2	15.4	0.4	2.5	4.5
**Spleen 1**	Mean value	89.3	175.8	30.0	450.4	772.8	38.6	24.5	681.0	13.6	801.7
	Standard deviation	6.7	2.6	0.0	14.4	16.6	0.7	2.2	1.6	0.4	6.6
	Standard deviation in %	7.5	1.5	0.1	3.2	2.1	1.7	9.1	0.2	3.0	0.8

Pork Tissues		Am	t^6A	i^6A	m^2_2G	m^2G	Q	OHyW	m^1G+Gm	m^6t^6A	m^1A
**Spleen 2**	Mean value	71.5	188.4	31.4	478.2	814.5	46.9	27.6	699.9	14.2	865.5
	Standard deviation	11.2	9.7	1.9	30.7	76.0	0.7	0.3	87.3	1.5	102.8
	Standard deviation in %	15.6	5.1	5.9	6.4	9.3	1.5	1.0	12.5	10.5	11.9
**Lung 1**	Mean value	47.0	178.1	31.6	408.0	847.2	25.9	20.7	773.7	13.2	864.9
	Standard deviation	1.2	4.3	0.1	5.3	3.2	4.6	0.7	0.1	1.2	3.7
	Standard deviation in %	2.5	2.4	0.3	1.3	0.4	17.6	3.5	0.0	8.8	0.4
**Lung 2**	Mean value	70.0	190.1	35.7	446.3	911.3	41.9	22.8	813.6	14.7	887.7
	Standard deviation	5.0	5.0	2.2	19.5	32.7	0.5	0.6	24.6	0.2	25.3
	Standard deviation in %	7.2	2.6	6.3	4.4	3.6	1.3	2.5	3.0	1.3	2.8
**Spine marrow 1**	Mean value	63.0	200.5	36.1	459.0	961.4	51.0	26.1	798.5	11.9	919.1
	Standard deviation	7.6	4.3	1.3	8.3	28.4	3.1	1.1	17.2	0.8	56.6
	Standard deviation in %	12.1	2.1	3.5	1.8	3.0	6.1	4.2	2.2	7.1	6.2
**Spine marrow 2**	Mean value	60.7	196.7	35.9	454.9	979.9	50.6	26.0	800.6	11.7	903.0
	Standard deviation	17.5	2.8	1.4	14.3	20.1	2.2	2.0	2.8	0.6	25.5
	Standard deviation in %	28.9	1.4	3.8	3.2	2.1	4.3	7.6	0.3	5.4	2.8
**Cerebellum 1**	Mean value	72.1	237.7	41.9	567.6	1,035.6	86.8	30.3	839.1	17.6	1,098.1
**Cerebellum 2**	Mean value	63.5	236.9	41.5	566.0	1,037.2	80.1	27.6	819.6	17.5	1,107.5
**Cerebrum 1**	Mean value	151.1	163.8	25.0	401.0	657.7	59.9	21.2	586.9	10.1	714.1
	Standard deviation	27.9	10.8	1.7	30.5	44.2	5.1	1.0	22.3	0.8	61.6
	Standard deviation in %	18.5	6.6	6.7	7.6	6.7	8.6	4.7	3.8	8.2	8.6

Pork Tissues		Am	t^6A	i^6A	m^2_2G	m^2G	Q	OHyW	m^1G+Gm	m^6t^6A	m^1A
**Cerebrum 2**	Mean value	128.7	167.8	27.2	411.3	706.7	54.6	19.5	586.7	11.0	751.2
	Standard deviation	0.1	7.8	0.7	6.1	19.0	1.5	0.7	18.7	0.4	38.1
	Standard deviation in %	0.1	4.6	2.5	1.5	2.7	2.8	3.8	3.2	3.8	5.1

Table S5. Modification numbers per 1000 tRNAs in different porcine tissues. The table lists the average values calculated from the modification content of at least two independent samples from one animal (with the exception of cerebellum, for which only one sample was analyzed). For each sample at least three independent digests and measurements were performed. 6.7 % mean standard deviation was obtained for all nucleosides excluding Am. 17.0 % mean standard deviation was obtained for Am.

Mouse Tissues		m^5C	Ψ
**Liver**	Mean value	1888.0	1986.9
	Standard deviation	68.3	49.4
	Standard deviation in %	3.6	2.5
**Cerebrum**	Mean value	1831.5	1639.2
	Standard deviation	84.2	61.8
	Standard deviation in %	4.6	3.8
**Lung**	Mean value	1747.8	1436.9
	Standard deviation	237.6	-
	Standard deviation in %	13.6	-
**Kidney**	Mean value	1316.5	924.5
	Standard deviation	81.8	65.6
	Standard deviation in %	6.2	7.1
**Spleen**	Mean value	1368.5	1284.7
	Standard deviation	98.1	2.7
	Standard deviation in %	7.2	0.2

Table S6. Modification numbers per 1000 tRNAs in different murine tissues. The table lists the average values calculated from the modification content of two sets of five animals (with the exception of cerebrum Ψ). For each sample at least two independent digests and measurements were performed. 5.4 % mean standard deviation was obtained for the two nucleosides. As noted in the Table S3, the overall mean standard deviation across nucleosides and tissues for the two individual sample sets is below 5 %.

Pork Tissues		m^1G	m^5C	Ψ
**Liver**	Mean value	746.7	2742.5	2263.1
	Standard deviation	3.2	253.6	347.0
	Standard deviation in %	0.4	9.2	15.3
**Spleen**	Mean value	627.1	2687.5	2146.1
	Standard deviation	7.8	182.1	161.5
	Standard deviation in %	1.2	6.8	7.5
**Kidney**	Mean value	628.8	2764.5	1886.8
	Standard deviation	10.6	217.0	270.2
	Standard deviation in %	1.7	7.9	14.3
**Lung**	Mean value	575.8	2452.7	1907.6
	Standard deviation	8.8	307.1	163.9
	Standard deviation in %	1.5	12.5	8.6
**Heart**	Mean value	535.1	2138.1	1776.1
	Standard deviation	3.1	269.5	203.0
	Standard deviation in %	0.6	12.6	11.4
**Cerebrum**	Mean value	448.5	1936.4	1330.4
		2.6	318.4	48.0
		0.6	16.4	3.6

Table S7. Modification numbers per 1000 tRNAs in different porcine tissues. The table lists the average values calculated from at least two independent digests and measurements. 7.3 % mean standard deviation was obtained for the three nucleosides. As noted in the Table S5, the overall mean standard deviation across nucleosides and tissues for individual sample sets is around 7 %.

Mouse tissues
Pork tissues

	average modification level	Am
Liver	1	0,135
Cerebrum	0,970	0,168
Liver	0,910	0,147
Cerebellum	0,900	0,485
Cerebellum	0,880	0,148
Lung	0,855	0,205
Brain stem	0,851	0,138
Spine marrow	0,746	0,442
Heart	0,713	0,188
Kidney	0,704	0,150
Kidney	0,694	0,489
Lung	0,691	0,418
Spleen	0,689	0,575
Spleen	0,676	0,342
Cerebrum	0,608	1
Heart	0,570	0,328

Table S8. Color-coded table listing the average modification levels and Am content in porcine and murine tissues. Values for each modification (listed in Fig. 2*A* and 2*B*) were normalized for the highest value across both organisms. The average modification level was calculated by averaging the normalized modification values.

Number	tRNA fragments	Liver (modified/unmodified)	Heart (modified/unmodified)
1	A-m^2G-Cp	1.00	0.62
2	G-m^2G-Up	0.49	0.41
3	m^1G-m^2G-Cp	0.59	0.35
4	t^6A-ACp	1.28	0.85
5	A-m^1A-AUp	20.3	3.69
6	G-m^1A-AACp	6.01	2.20
7	A-i^6A-ACp	1.07	0.14
8	G-m^1A-GCp	0.25	0.24
9	G-m^1A-Up	0.27	0.28
10	A-ms^2i^6A-AGCp	Traces (<0.1)	0.33

Table S9. Representative modified tRNA fragments analyzed after RNase A digestion of porcine tRNA, calculated using Sprinzl tRNA database [8] sequences and the Mongo Oligo Mass Calculator program (http://library.med.utah.edu/masspec/mongo.htm). The mass area ratios of modified to unmodified fragments from total tRNA of liver and heart are shown using the z=-2 peak. Analyzed fragments 1-7 clearly represent higher modification in liver than in heart. Fragments 8 and 9 have similar modified to unmodified ratios. Fragment 10 represents a mitochondrial tRNA fragment containing m^2i^6A, which is present in heart and only in traces in liver. These relative non-quantitative data are in strict accordance to the quantitative values described in Fig. 2.

**Intra-assay**	A(m^{1}A)	A([D$_3$]m^{1}A)	m^{1}A /1000 tRNAs	A(i^{6}A)	A([D$_2$]i^{6}A)	i^{6}A /1000 tRNAs
1	4,910,905	3,228,128	231.7	20,679,891	24,434,161	81.5
2	4,610,522	3,000,111	234.1	22,055,338	26,075,813	81.5
3	4,519,200	2,977,821	231.1	22,851,651	26,747,503	82.3
4	4,296,971	2,795,390	234.1	22,336,638	26,299,470	81.8
5	4,256,358	2,959,025	218.9	23,127,819	27,224,872	81.8
Mean value	4,518,791	2,992,095	230.0	22,210,267	26,156,364	81.8
RSD %	5.2	4.6	2.5	3.8	3.6	0.4

**Intra-assay**	A(ms^{2}i^{6}A)	A([D$_3$]ms^{2}i^{6}A)	ms^{2}i^{6}A /1000 tRNAs	A(m^{1}G)	A([D$_3$]m^{1}G)	m^{1}G /1000 tRNAs
1	7,565,152	6,021,535	34.4	1,555,016	1,882,259	135.5
2	8,516,277	6,814,035	34.2	1,629,176	1,901,322	140.8
3	8,736,921	6,919,317	34.6	1,564,160	1,880,098	136.5
4	8,595,797	6,922,770	34.0	1,530,903	1,860,304	134.9
5	9,057,064	7,301,879	34.0	1,614,978	1,842,588	144.2
Mean value	8,494,242	6,795,907	34.3	1,578,847	1,873,314	138.4
RSD %	5.9	6.2	0.7	2.4	1.1	2.6

**Inter-assay**	A(m^{1}A)	A([D$_3$]m^{1}A)	m^{1}A /1000 tRNAs	A(i^{6}A)	A([D$_2$]i^{6}A)	i^{6}A /1000 tRNAs
1	5,203,410	3,331,919	237.9	14,545,585	17,095,570	82.0
2	2,987,553	1,958,336	232.3	12,575,227	14,579,015	83.2
3	3,225,581	2,095,535	234.4	12,151,803	14,540,493	80.4
4	1,951,525	1,265,851	234.8	10,028,667	11,761,222	82.2
5	4,256,358	2,959,025	218.9	23,127,819	27,224,872	81.8
6	3,249,988	2,223,235	222.5	21,114,201	24,952,572	81.5
Mean value	3,479,069	2,305,650	230.1	15,590,550	18,358,957	81.8
RSD %	29.4	29.3	6.3	31.0	31.1	1.0

**Inter-assay**	A(ms^{2}i^{6}A)	A([D$_3$]ms^{2}i^{6}A)	ms^{2}i^{6}A /1000 tRNAs	A(m^{1}G)	A([D$_3$]m^{1}G)	m^{1}G /1000 tRNAs
1	5,974,676	4,846,268	33.7	926,498	1,054,171	144.6
2	5,461,095	4,327,040	34.6	705,140	894,683	128.9
3	3,881,613	3,101,702	34.3	641,656	776,294	135.5
4	3,758,364	2,908,332	35.5	439,096	544,671	132.0
5	9,057,064	7,301,879	34.0	1,614,978	1,842,588	144.2
6	8,707,594	6,786,037	35.2	1,452,283	1,713,325	139.2
Mean value	6,140,068	4,878,543	34.5	963,275	1,137,622	137.4
RSD %	34.1	34.4	1.8	44.7	42.1	4.3

Table S10. Intra- and Inter-assay test of representative nucleosides m^{1}A, i^{6}A, ms^{2}i^{6}A and m^{1}G.

Data set	Mean	Standard deviation	n	Shapiro Wilk W	Shapiro Wilk significance
Normalized murine nucleosides	0.87	0.15	4	0.90	0.46
Normalized porcine nucleosides	0.75	0.13	6	0.85	0.15
Protein synthesis rate (this study, all murine tRNA)	0.38	0.29	11	0.94	0.52
Protein synthesis rate (this study, cytosolic porcine tRNA)	0.75	0.24	6	0.93	0.57
Protein synthesis rate (this study, all porcine tRNA)	0.84	0.13	6	0.96	0.82
Protein synthesis rate (this study, all porcine tRNA excluding heart)	0.83	0.15	5	0.95	0.70
Protein Synthesis rate (Suryawan, %/d)	35.5	20.5	5	0.878	0.26

Table S11: Descriptive Statistics. All calculations were carried out using the SPSS or Statistica statistics programs. Correlations are calculated as Pearson's r coefficients with 2-tailed significance values. This test was chosen as the data sets used in correlations appear to be parametric as evidenced by non-significant scores in the Shapiro-Wilk test. The small sample sizes are recognized as a possible source of error and correspondingly it cannot be excluded that more complex relationships than simple linear correlations may be present.

Protein synthesis rate	r	Significance (P value)	n
This study (cytosolic porcine tRNA)	0.861*	0.028	6
This study (all porcine tissues, total tRNA)	0.672	0.143	6
This study (all porcine tissues except heart, total tRNA)	0.965**	0.008	5
This study (all murine tissues, total tRNA)	0.723*	0.011	11
Suryawan	0.956*	0.011	5

Table S12. Correlations of normalized nucleoside levels (excluding ms^2i^6A) with protein synthesis rates. * significant to 5 % level, ** significant to 1 % level.

Supplementary Information References

[1] T. Brückl, D. Globisch, M. Wagner, M. Müller, T. Carell, *Angew. Chem. Int. Ed.* **2009**, *48*, 7932-7934.

[2] D. Globisch, D. Pearson, A. Hienzsch, T. Brückl, M. Wagner, I. Thoma, P. Thumbs, V. Reiter, A. C. Kneuttinger, M. Müller, S. A. Sieber, T. Carell, *Angew. Chem. Int. Ed.* **2011**, *50*, 9739-9742.

[3] V. Reiter, D. M. S. Matschkal, M. Wagner, D. Globisch, A. C. Kneuttinger, M. Müller, T. Carell, *Nucleic Acids Res.* **2012**, *40*, 6235-6240.

[4] R. J. Jackson, S. Napthine, I. Brierley, *RNA* **2001**, *7*, 765-773.

[5] J. B. Plotkin, H. Robins, A. J. Levine, *Proc. Natl. Acad. Sci. U S A* **2004**, *101*, 12588-12591.

[6] A. Suryawan, P. M. J. O'Connor, J. A. Bush, H. V. Nguyen, T. A. Davis, *Amino Acids* **2009**, *37*, 97-104.

[7] C. T. Y. Chan, M. Dyavaiah, M. S. DeMott, K. Taghizadeh, P. C. Dedon, T. J. Begley, *PLoS Genet.* **2010**, *6*, e1001247.

[8] F. Jühling, M. Mörl, K. Hartmann Roland, M. Sprinzl, F. Stadler Peter, J. Pütz, *Nucleic Acids Res.* **2009**, *37*, D159-D162.
